# Factors Related to Blood Loss During Endoscopic Sinus Surgery

**DOI:** 10.7759/cureus.76286

**Published:** 2024-12-23

**Authors:** Yamato Oki, Hiromitsu Hatakeyama, Kouzi Yamamoto, Kenta Hukui, Shota Yamada, Kizima Natsumi, Tai Iwamura, Yu Matsumoto, Kaname Sato, Nobuhiko Oridate

**Affiliations:** 1 Otolaryngology, Yokohama City University Medical Center, Yokohama, JPN; 2 Clinical Statistics, Yokohama City University Graduate School of Medicine, Yokohama, JPN; 3 Otolaryngology, Yokohama City University Graduate School of Medicine, Yokohama, JPN

**Keywords:** blood loss, endoscopic sinus surgery, ess, lund-mackay score, pt-inr

## Abstract

Background: The safety and efficacy of endoscopic sinus surgery have improved with the development of new equipment and improved surgical techniques. However, it is accompanied by the risk of complications. Intraoperative blood loss is an important factor in the safe conduct of surgery. Therefore, we examined the factors associated with intraoperative blood loss.

Method: The amount of intraoperative bleeding experienced by 518 patients with sinonasal disease who underwent endoscopic sinus surgery under general anaesthesia at our hospital over nine years was tabulated. Thirty-four variables were extracted after analysis of patients' background, sinonasal pathology, and haematology results. Multivariate linear regression analysis was performed.

Results: Multivariate analysis revealed significant differences in the prothrombin time-international normalized ratio (PT-INR), Lund-Mackay score, operative time, and initial versus repeat surgery. The degree of change between the 25% and 75% points was significant for the following variables: an increase from 5 to 14 points for the Lund-Mackay score at 73 mL.

Conclusions: Preoperative precautionary measures should be implemented in the event of re-operation, expected prolonged operative time, high Lund-Mackay score, and prolonged PT-INR. Intraoperative blood loss in patients with a high Lund-Mackay score requires meticulous attention.

## Introduction

Endoscopic sinus surgery (ESS) is the gold standard for the treatment of chronic rhinosinusitis (CRS) [1,2]. In recent years, the safety and efficacy of ESS have improved with the development of new equipment and improvement in surgical techniques [2]. However, there still exists an attendant risk of complications associated with orbital or skull base injury, such as spinal fluid fistula and optic nerve damage [3]. The success or failure of ESS depends on numerous factors, and contamination of the surgical field due to excessive bleeding constitutes one of the most important factors [4]. Excessive blood loss is sometimes difficult to predict and manage [5]. Intraoperative blood loss is an important factor for the safe performance of ESS [6], but few studies have conducted an extensive examination of the factors related to blood loss in ESS. In this study, we examined the factors associated with intraoperative blood loss during ESS at our hospital.

This article was previously posted to the medRxiv preprint server on April 27, 2020.

## Materials and methods

Patients

The study was conducted at Yokohama City University Medical Center, Yokohama City, Kanagawa Prefecture, Japan. The amount of intraoperative bleeding experienced by 518 patients with sinonasal disease who underwent ESS under general anaesthesia at this hospital between April 2012 and March 2020 was tabulated. Patients with neoplastic lesions and those who required extra-nasal incision were excluded.

The amount of bleeding was measured by subtracting the amount of saline solution sprayed into the nose to clean the surface of the lens from that aspirated by the suction tube and microdebrider during ESS.

Factors for analysis

A total of 34 variables were extracted for analysis in this study as follows: patient background (age, sex, hypertension, asthma, antithrombotic medication, smoking habit, alcohol consumption, preoperative systolic blood pressure (sBP), and preoperative diastolic blood pressure (dBP)), nasal sinus pathology (sinus disease, Lund-Mackay score, number of inflamed sinuses, nasal polyps, preoperative macrolide use, preoperative steroid use, operative time, initial surgery or re-operation, unilateral or bilateral, and number of open sinuses), and blood test results (white blood cell (WBC), neutrophil/lymphocyte (Neu/Lym) ratio, eosinophils (Eos), haemoglobin (Hb), platelet (Plt), albumin (Alb), aspartate aminotransferase (AST), alanine aminotransferase (ALT), γ-glutamyl transpeptidase (γ-GTP), alkaline phosphatase (ALP), total bilirubin (T-bil), creatinine, C-reactive protein (CRP), prothrombin time-international normalized ratio (PT-INR), and activated partial thromboplastin time (APTT)).

Criteria for analysis

The participants were stratified into 11 groups according to bleeding volume in increments of 50 mL. The variance inflation factor was calculated for each study variable to confirm multicollinearity. Multivariate analysis by linear regression was performed, and standardized regression coefficients were calculated for each study variable. The confidence intervals and the amount of change between the 25% and 75% points were calculated.

The analyses were performed by a clinical statistician at our hospital. All statistical analyses were performed using the statistical analysis software R (R Core Team, 2021). P-values less than 0.05 were considered statistically significant.

This study was approved by the Research Ethics Review Committee of Yokohama City University Medical Center (approval number: B210500054).

## Results

Bleeding volume and stratification

**Figure 1:**
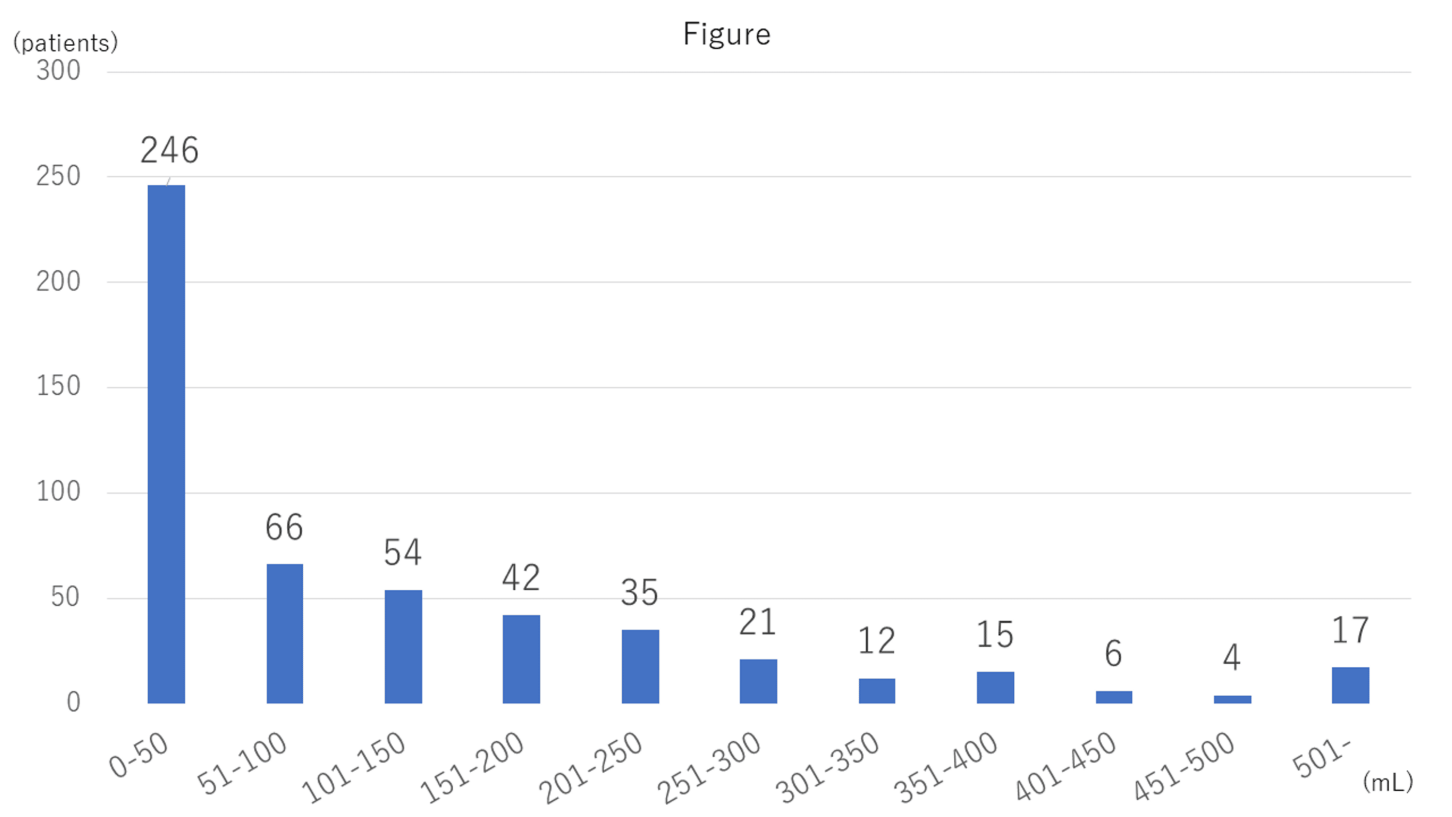
Intraoperative blood loss was measured and stratified in increments of 50 mL. The horizontal axis indicates the amount of blood loss, and the vertical axis indicates the number of patients. Nearly half of the patients lost between 0 and 50 mL of blood. The number of patients in each group decreased gradually with the increase in blood loss and rose in the heaviest bleeding group, i.e., blood loss of 500 mL or more.

Intraoperative blood loss was measured and stratified in increments of 50 mL. Nearly half of the patients lost between 0 and 50 mL of blood. The number of patients in each group decreased gradually with increasing blood loss, with a rise in the heaviest bleeding group (500 mL or more) (Figure 1).

Patient characteristics

The patient characteristics are shown in Table 1 and Table 2. There was a slight male predominance, and while variability was observed across all items, no items showed significant deviations from the mean or median.

**Table 1:** The factors analysed in this study about patient characteristics. sBP: systolic blood pressure; dBP: diastolic blood pressure

Analysis items (n=518)	Value	Median
Age (years)	6-88	53
Preoperative sBP (mmHg)	79-179	122
Preoperative dBP (mmHg)	32-116	74
Number of inflamed sinuses (pieces)	0-10	6
Lund-Mackay score (points)	0-24	8
Operative time (minutes)	20-349	154
Number of sinuses opened (pieces)	1-10	8

**Table 2:** The factors analysed in this study about patient characteristics. CRS: chronic rhinosinusitis

Analysis items (n=518)	Number	Ratio
Sex (man)	343	66.2%
Hypertension (yes)	126	24.3%
Asthma (yes)	122	23.5%
Antithrombotic medication (yes)	23	4.4%
Smoking habit (yes)	261	50.9%
Alcohol consumption (yes)	282	54.3%
Sinus disease		
Odontogenic maxillary sinusitis	61	11.8%
Fungal sinusitis	53	10.2%
Eosinophilic CRS	65	12.5%
Cystic disease	24	4.6%
CRS with/without nasal polyps	315	60.7%
Nasal polyps (yes)	309	59.5%
Preoperative macrolide dose (yes)	162	31.2%
Preoperative steroid dose (yes)	63	12.1%
Initial surgery or re-operation (initial)	428	82.6%
Unilateral or bilateral (bilateral)	313	60.4%

Sinonasal pathology

The sinonasal pathology is shown in Table 1 and Table 2. A variety of diseases were represented without any significant predominance of a particular disease. There was considerable variability in the extent and density of sinusitis, with the overall presentation considered average. Preoperative use of antibiotics and steroids for anti-inflammatory purposes was observed.

Surgical factors

The surgical factors are shown in Table 1 and Table 2. There was wide variation in operative time among individual cases. While most patients underwent their first surgery, some had previous surgical histories. Given the nature of the diseases, postoperative maxillary cysts and eosinophilic CRS (ECRS) were frequently observed. The extent of the surgical field was determined by the size and location of the lesion, leading to variation in the number of openings created.

Laboratory blood data

The laboratory blood data is shown in Table 3. There was considerable variability in the blood test results. While the median values for most parameters fell within the normal range, there was a wide distribution of data.

**Table 3:** The factors analysed in this study about laboratory blood data. WBC: white blood cell; Neu/Lym: neutrophil/lymphocyte; Eos: eosinophils; Hb: haemoglobin; Plt: platelet; Alb: albumin; AST: aspartate aminotransferase; ALT: alanine aminotransferase; γ-GTP: γ-glutamyl transpeptidase; ALP: alkaline phosphatase; T-bil: total bilirubin; CRP: C-reactive protein; PT-INR: prothrombin time-international normalized ratio; APTT: activated partial thromboplastin time

Analysis items (n=518)	Value	Median
WBC (/μl)	2600-12830	6005
Neu/Lym ratio	0.64-19.4	1.82
Eos (%)	0.1-59.1	3.65
Hb (g/dl)	9.3-17.8	14.3
Plt (10^4^/μl)	10.1-50.1	23.6
Alb (g/dl)	2.8-5.6	4.5
AST (U/L)	9-164	22
ALT (U/L)	6-169	19
γ-GTP (U/L)	8-213	25
ALP (U/L)	96-1837	218
T-bil (mg/dl)	0.3-2.7	0.7
Creatinine (mg/dl)	0.34-12.61	0.75
CRP (mg/dl)	0.001-11.319	0.104
PT-INR	0.81-3.97	0.97
APTT (seconds)	13.7-72	30.3

Variance inflation factors

The results of the analysis of the coefficient of variance are shown in Table 4. None of the variables exhibited sufficient multicollinearity to cause problems in the multivariate analysis.

**Table 4:** Variance inflation factors for the variables analysed in this study. None of the variables possessed sufficient multicollinearity to cause problems in the multivariate analysis. sBP: systolic blood pressure; dBP: diastolic blood pressure; CRS: chronic rhinosinusitis; WBC: white blood cell; Neu/Lym: neutrophil/lymphocyte; Eos: eosinophils; Hb: haemoglobin; Plt: platelet; Alb: albumin; AST: aspartate aminotransferase; ALT: alanine aminotransferase; γ-GTP: γ-glutamyl transpeptidase; ALP: alkaline phosphatase; T-bil: total bilirubin; CRP: C-reactive protein; PT-INR: prothrombin time-international normalized ratio; APTT: activated partial thromboplastin time

Analysis items (n=518)	Variance inflation factors
Sex (man)	1.79
Age (years)	1.94
Hypertension (yes)	2.06
Asthma (yes)	1.51
Antithrombotic medication (yes)	1.19
Smoking habit (yes)	1.36
Alcohol consumption (yes)	1.28
Preoperative sBP (mmHg)	2.41
Preoperative dBP (mmHg)	1.12
Sinus disease	
Odontogenic maxillary sinusitis	3.08
Fungal sinusitis	2.92
Cystic disease	2.16
CRS with/without nasal polyps	3.62
Number of inflamed sinuses (pieces)	5.47
Lund-Mackay score (points)	5.4
Nasal polyps (yes)	1.79
Preoperative macrolide use (yes)	1.16
Preoperative steroid use (yes)	1.34
Operative time (minutes)	1.72
Initial surgery or re-operation (re)	1.41
Unilateral or bilateral (bilateral)	4.27
Number of sinuses opened (pieces)	7.15
WBC (/μl)	1.51
Neu/Lym ratio	1.33
Eos (%)	1.41
Hb (g/dl)	2.26
Plt (10^4^/μl)	1.48
Alb (g/dl)	1.64
AST (U/L)	3.05
ALT (U/L)	3.73
γ-GTP (U/L)	1.6
ALP (U/L)	1.21
T-bil (mg/dl)	1.29
Creatinine (mg/dl)	1.1
CRP (mg/dl)	1.5
PT-INR	1.24
APTT (seconds)	1.12

Multivariate analysis by linear regression

Multivariate analysis (Table 5) revealed significant differences (p<0.05) in PT-INR, the Lund-Mackay score, operative time, and history of surgery. No significant differences were found for the other variables.

**Table 5:** Results of multivariate analysis by linear regression. Significant (p<0.05) differences were observed in PT-INR, Lund-Mackay score, operative time, and initial surgery versus re-operation. No significant differences were found for the other items. sBP: systolic blood pressure; dBP: diastolic blood pressure; CRS: chronic rhinosinusitis; WBC: white blood cell; Neu/Lym: neutrophil/lymphocyte; Eos: eosinophils; Hb: haemoglobin; Plt: platelet; Alb: albumin; AST: aspartate aminotransferase; ALT: alanine aminotransferase; γ-GTP: γ-glutamyl transpeptidase; ALP: alkaline phosphatase; T-bil: total bilirubin; CRP: C-reactive protein; PT-INR: prothrombin time-international normalized ratio; APTT: activated partial thromboplastin time *p<0.05; **p<0.01; ***p<0.001

Analysis items (n=518)	Estimate	Standardized	Std. error	Pr(>|t|)
Sex (woman)	-22.499	-0.085	14.183	0.11
Age (years)	-0.551	-0.076	0.409	0.18
Hypertension (yes)	-1.795	-0.0062	16.798	0.91
Asthma (yes)	-16.839	-0.057	14.573	0.25
Antithrombotic medication (yes)	-29.816	-0.049	26.499	0.26
Smoking habit (yes)	7.467	0.030	11.711	0.52
Alcohol consumption (yes)	-1.397	-0.0056	11.379	0.90
Preoperative sBP (mmHg)	0.0072	0.0011	0.403	0.99
Preoperative dBP (mmHg)	-0.148	-0.047	0.133	0.27
Sinus disease				
Odontogenic maxillary sinusitis	15.940	0.041	27.463	0.56
Fungal sinusitis	23.230	0.057	28.232	0.41
Cystic disease	-30.639	-0.052	35.002	0.38
CRS with/without nasal polyps	-13.456	-0.053	19.594	0.49
Number of inflamed sinuses (pieces)	2.260	0.054	3.947	0.57
Lund-Mackay score (points)	-8.116	0.385	1.973	<0.0001
Nasal polyps (yes)	1.807	0.0071	13.690	0.90
Preoperative macrolide dose (yes)	1.688	0.0063	11.653	0.88
Preoperative steroid dose (yes)	-13.475	-0.035	17.754	0.45
Operative time (minutes)	0.286	0.145	0.104	0.0063
Initial surgery or re-operation (re)	49.098	0.150	15.690	0.0019
Unilateral or bilateral (bilateral)	-0.689	-0.0027	21.204	0.97
Number of sinuses opened (pieces)	-2.680	-0.072	3.983	0.50
WBC (/μl)	0.0070	0.096	0.004	0.05
Neu/Lym ratio	1.321	0.015	4.181	0.75
Eos (%)	-1.364	-0.052	1.254	0.28
Hb (g/dl)	4.185	0.045	5.579	0.28
Plt (10^4^/μl)	-1.586	-0.075	1.038	0.13
Alb (g/dl)	-16.818	-0.045	19.300	0.38
AST (U/L)	-0.014	-0.0013	0.774	0.99
ALT (U/L)	0.216	0.026	0.635	0.73
γ-GTP (U/L)	-0.085	-0.020	0.211	0.69
ALP (U/L)	-0.039	-0.040	0.043	0.36
T-bil (mg/dl)	-22.763	-0.051	20.299	0.26
Creatinine (mg/dl)	-0.906	-0.004	9.626	0.93
CRP (mg/dl)	-3.138	-0.019	8.147	0.70
PT-INR	64.883	0.090	32.356	0.046
APTT (seconds)	1.298	0.049	1.131	0.25

The confidence intervals and the degree of change between the 25% and 75% points are shown in Table 6. The four principal factors that showed significant differences were an increase of 1 in the PT-INR resulting in a 64.9 mL increase, an increase in the Lund-Mackay Score from 5 to 14 points corresponding to a 73.0 mL increase, an increase in operative time from 102 to 201 minutes corresponding to a 28.2 mL increase, and an increase in the frequency of re-operation versus initial surgery corresponding to a 49.1 mL increase.

**Table 6:** Confidence intervals and the amount of change between the 25% and 75% points. The four main items that showed significant differences were as follows: an increase of 1 in PT-INR at 64.9 mL, an increase in the Lund-Mackay score from 5 to 14 points at 73 mL, an increase in operative time from 102 to 201 minutes to 28.2 mL, and an increase in the frequency of re-operation compared to initial surgery at 49.1 mL. sBP: systolic blood pressure; dBP: diastolic blood pressure; CRS: chronic rhinosinusitis; WBC: white blood cell; Neu/Lym: neutrophil/lymphocyte; Eos: eosinophils; Hb: haemoglobin; Plt: platelet; Alb: albumin; AST: aspartate aminotransferase; ALT: alanine aminotransferase; γ-GTP: γ-glutamyl transpeptidase; ALP: alkaline phosphatase; T-bil: total bilirubin; CRP: C-reactive protein; PT-INR: prothrombin time-international normalized ratio; APTT: activated partial thromboplastin time

Analysis items (n=518)	Low	High	Effect	95% CI
Sex (woman)	1.000	2.000	-22.499	-50.369 to 5.370
Age (years)	41.000	66.750	-14.194	-34.897 to 6.510
Hypertension (yes)	1.000	2.000	-1.795	-34.802 to 31.210
Asthma (yes)	1.000	2.000	-16.839	-45.474 to 11.800
Antithrombotic medication (yes)	1.000	2.000	-29.816	-81.885 to 22.250
Smoking habit (yes)	2.000	1.000	-7.467	-30.480 to 15.540
Alcohol consumption (yes)	2.000	1.000	1.397	-20.963 to 23.760
Preoperative sBP (mmHg)	109.000	136.000	0.195	-21.182 to 21.570
Preoperative dBP (mmHg)	65.000	85.000	-2.961	-8.194 to 2.270
Sinus disease (vs CRS w/ sNP)				
Odontogenic maxillary sinusitis	5.000	2.000	29.396	-9.074 to 67.870
Fungal sinusitis	5.000	4.000	36.686	-4.150 to 77.520
Cystic disease	5.000	3.000	-17.183	-75.246 to 40.880
Eosinophilic CRS	5.000	1.000	13.456	-25.046 to 51.960
Number of inflamed sinuses (pieces)	0.000	10.000	-18.759	-73.544 to 36.030
Lund-Mackay score (points)	5.000	14.000	73.047	38.151 to 107.940
Nasal polyps (yes)	2.000	1.000	-1.807	-28.706 to 25.090
Preoperative macrolide dose (yes)	1.000	2.000	1.688	-21.209 to 24.590
Preoperative steroid dose (yes)	1.000	2.000	-13.475	-48.361 to 21.410
Operative time (minutes)	102.000	201.000	28.229	8.013 to 48.450
Initial surgery or re-operation (initial)	1.000	2.000	49.098	18.268 to 79.930
Unilateral or bilateral (bilateral)	2.000	1.000	0.689	-40.976 to 42.350
Number of sinuses opened (pieces)	4.000	10.000	13.561	-32.971 to 60.090
WBC (/μl)	5100.000	7292.500	15.407	-0.199 to 31.010
Neu/Lym ratio	1.000	2.449	1.412	-7.369 to 10.190
Eos (%)	1.000	6.400	-6.272	-17.604 to 5.060
Hb (g/dl)	13.000	15.100	7.846	-12.708 to 28.400
Plt (10^4^/μl)	19.000	27.375	-12.013	-27.455 to 3.430
Alb (g/dl)	4.000	4.700	-6.727	-21.897 to 8.440
AST (U/L)	18.000	27.000	-0.124	-13.818 to 13.570
ALT (U/L)	13.000	28.750	3.407	-16.244 to 23.060
γ-GTP (U/L)	16.000	42.000	-2.198	-12.956 to 8.560
ALP (U/L)	185.000	278.750	-3.685	-11.661 to 4.290
T-bil (mg/dl)	0.000	0.800	-4.553	-12.530 to 3.420
Creatinine (mg/dl)	0.000	0.860	-0.199	-4.360 to 3.960
CRP (mg/dl)	0.000	0.223	-0.548	-3.346 to 2.250
PT-INR	0.000	1.020	5.839	0.117 to 11.560
APTT (seconds)	28.000	1.020	5.809	-4.135 to 15.750

## Discussion

Several studies have focused on excessive bleeding control in ESS, the endoscopic view, and methods for bleeding control. These studies examined the appropriate methods to reduce blood loss and make the procedure easier and safer [1-25]. We think that identifying the risk factors for excessive bleeding will help clinicians focus on bleeding concerns and plan and implement countermeasures in advance. The factors that showed significant differences were operative time, initial surgery versus re-operation, Lund-Mackay score, and PT-INR. Because the degree of the 25% and 75% points was the largest in the Lund-Mackay score at 73 mL, this score exhibited the strongest correlation with blood loss. Previous studies have demonstrated that extensive lesions associated with CRS often correlate with excessive intraoperative bleeding [7,8]. Our findings are consistent with these reports. On the other hand, the number of sinuses opened by surgery, the number of inflamed sinuses, and the laterality of the operative site (bilateral or unilateral) were not independent factors. For example, in the case of a single sinus lesion of the sphenoid sinus, the intact ethmoidal or maxillary sinus may be opened if necessary. The present analysis suggests that opening sinuses with no or little inflammation do not or are unlikely to affect the amount of bleeding. In summary, we found that the amount of bleeding is correlated with the severity of sinusitis and not with the extent of surgery and that the Lund-Mackay score is a good system that reflects the severity of sinusitis.

Furthermore, surgical duration was identified as an independent factor contributing to increased intraoperative blood loss. However, the operative time can only be evaluated retrospectively owing to its inherent nature. Therefore, it is difficult to determine whether the surgery takes longer because of excessive blood loss or whether a longer operative time results in greater blood loss. Previous studies reported that the total amount of bleeding depends directly on the duration of surgery [9,10]. We think that the fact that operative time was deemed to be an independent factor is important for this study's accuracy since it coincides with the results of previous analyses.

PT-INR, which was found to be independently correlated with the amount of bleeding in this study, is an indicator of coagulation capacity. It is obvious that the higher the PT-INR value, the greater the bleeding volume.

The amount of blood loss was greater in patients undergoing re-operation compared to those undergoing ESS for the first time. Surgical treatment is more difficult in recurrent cases due to scarring and changes in nasal morphology. Moreover, patients with ECRS are prone to recurrence [11], and some Japanese reports suggested that ESS of ECRS usually causes more blood loss than other sinus diseases [12]. In the English literature, few studies exist that focus on the concept of ECRS, and some researchers have stated that the intensity of local inflammation affects bleeding during ESS [9]. Moreover, blood loss presents a substantial problem during ESS in patients with broad nasal polyps [9]. ECRS is characterized by prominent bilateral nasal polyps and intense eosinophilic inflammation [11]; thus, it can be inferred that patients with ECRS are prone to excessive bleeding during ESS. The possible reasons for the high incidence of bleeding in recurrent cases include scar formation, postoperative changes, and a high degree of local inflammation, especially in cases with frequent recurrence.

Additionally, points expected to be related to blood loss but not identified as independent factors are also discussed.

The amount of bleeding did not differ significantly with respect to the type of sinus disease. As a tendency, the amount of bleeding was lesser in cystic disease and greater in odontogenic maxillary sinusitis and fungal sinusitis. On the other hand, the proportion of recurrent cases was 22.6% in cystic disease with a higher frequency of postoperative maxillary cysts, 54.8% in CRS, and 19.4% in ECRS. The proportion of cystic disease and ECRS was large compared to overall, but it did not agree with the data on bleeding tendency by disease. In addition to the factors that re-operation itself has, there might be other factors that increase bleeding in a group of patients with recurrence. Analysis with a different study design is needed for the next.

None of the items related to hypertension were statistically significant. In addition to a history of hypertension, some patients presented with high blood pressure on the day before surgery, which was not correlated with excessive bleeding. We believe that the systemic management for general anaesthesia played a major role in these results. In our hospital, we often ask the anaesthesiologist to manage the sBP so that it does not exceed 100 mmHg. Previous studies have also shown that controlling the mean arterial pressure and heart rate facilitates the management of bleeding and secures the visual field [13]. This result suggests the importance of good communication with the anaesthesiologist during surgery to maintain the blood pressure and heart rate under control.

WBC and CRP are widely used haematological indicators of inflammation. However, no significant differences were found in these factors with respect to intraoperative blood loss in the present analysis. In the case of chronic sinusitis, it is possible that the inflammation was only local and did not extend systemically. In fact, the WBC and CRP levels were within the normal range in most cases. The degree of inflammation on computed tomography and nasal examination may show a better correlation compared to the severity of inflammation on haematological testing.

The main limitations of this study are outlined below.

While the quantification of blood loss provides an objective method for assessing intraoperative bleeding [14,15], it has been criticized as an accurate measurement approach because not only blood but also tissue and irrigation fluids are collected in suction bottles intended for blood collection [16]. Nevertheless, most studies have measured blood volume using suction systems [17-25]. In our analysis, we mitigated the impact of measurement bias by categorizing patients into 50 mL increments based on blood loss in each case.

Another limitation is the retrospective design of the study. While the large number of cases reflects the extended timeframe over which data were collected, there was a lack of standardization among the surgeons, even though the instructors remained consistent. Furthermore, the method of general anaesthesia varied depending on patient-specific factors, encompassing both inhalation and intravenous techniques. Although the retrospective approach was suitable for analysing surgical blood loss, future studies should aim to minimize potential biases by standardizing key variables such as surgeons, surgical techniques, and anaesthesia protocols.

## Conclusions

We examined the factors that correlated with the amount of blood loss during ESS at our hospital. The results showed that preoperative precautionary measures should be implemented in the event of re-operation, expected long operative time, high Lund-Mackay score, and prolonged PT-INR. Particular attention should be paid to intraoperative blood loss in patients with a high Lund-Mackay score.
